# An ab initio study of the magnetic properties of strontium hexaferrite

**DOI:** 10.1038/s41598-021-81028-7

**Published:** 2021-01-21

**Authors:** C. Tejera-Centeno, S. Gallego, J. I. Cerdá

**Affiliations:** Instituto de Ciencia de Materiales de Madrid, CSIC, Cantoblanco, 28049 Madrid Spain

**Keywords:** Theory and computation, Magnetic properties and materials

## Abstract

The magnetic properties of ${\text{SrFe}}_{12}{\text{O}}_{19}$, a paradigmatic hexaferrite for permanent magnet applications, have been addressed in detail combining density functional theory including spin–orbit coupling and a Hubbard U term with Monte Carlo simulations. This multiscale approach allows to estimate the Néel temperature of the material from ab initio exchange constants, and to determine the influence of different computational conditions on the magnetic properties by direct comparison versus available experimental data. It is found that the dominant influence arises from the choice of the Hubbard U term, with a value in the 2–3 eV range as the most adequate to quantitatively reproduce the two most relevant magnetic properties of this material, namely: its large perpendicular magnetocrystalline anisotropy and its elevated Néel temperature.

## Introduction

Hexagonal ferrites, or hexaferrites, are hard ferrimagnets that find application as permanent magnets in magnetic- and magneto-optical recording, or as components in electrical devices, representing the bulk of the total magnetic materials manufactured globally. Although their performance is below that of rare-earth-based permanent magnets, they bring important advantages due to their abundance and chemical stability, high Néel temperatures, easy preparation and low cost^1^.

**Figure 1:**
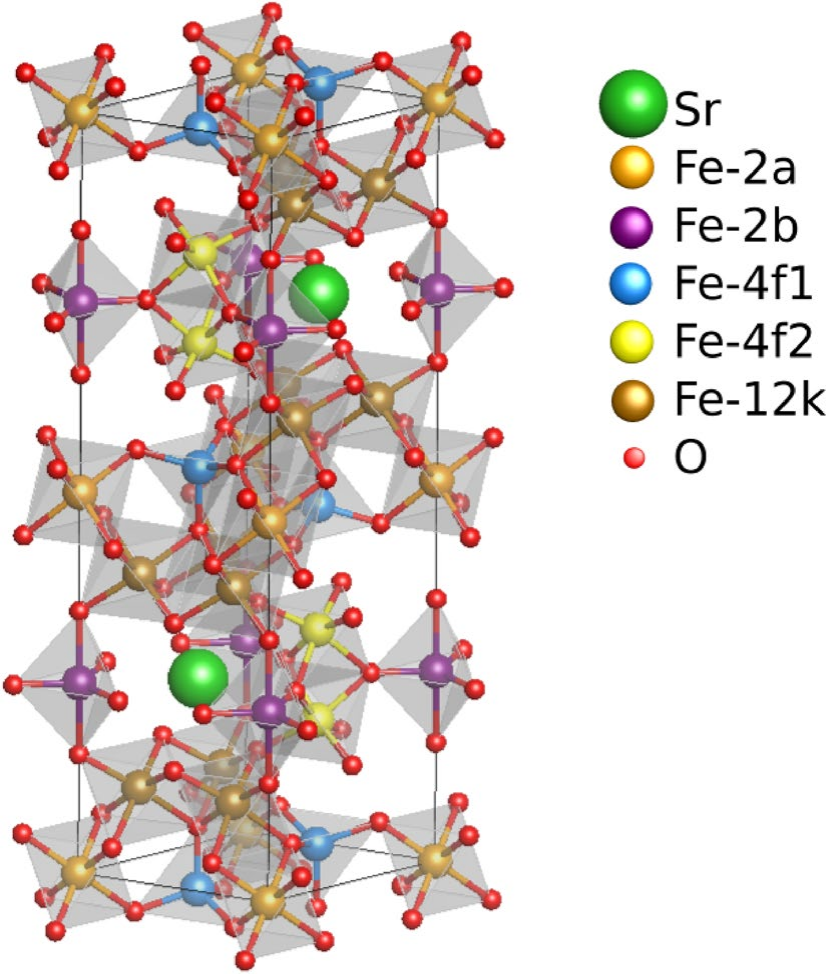
Atomic structure of the SFO. The unit cell contains two formula units, $({\text{SrFe}}_{12}{\text{O}}_{19})_2$, and has 64 atoms in total. The environement around each Fe ion has been shaded in order to highlight its coordination to oxygen atoms (tetrahedral, octahedral or bipyramidal). This figure has been obtained using VESTA^20^ version 3.5.1 (https://jp-minerals.org/vesta/en/).

Among them, SFO (${\text{SrFe}}_{12}{\text{O}}_{19}$) is particularly attractive, due to its large saturation magnetization and coercivity, a large uniaxial magnetocrystalline anisotropy (MCA) and excellent chemical stability. It is an *M*-type hexaferrite isostructural to magnetoplumbite with space group $P6_{3/mmc}$. The SFO structure, shown in Fig. 1, has five different sublattices: 2*a*, 2*b*, 12*k* with parallel and 4*f*1, 4*f*2 with antiparallel magnetic moments (MMs), what makes it a ferrimagnet. Sublattices 2*a*, 12*k* and 4*f*2 have an octahedral environment (the latter being distorted due to the presence of the Sr atoms), 2*b* a bipyramidal one while 4*f*1 is tetrahedrally coordinated. The magnetic coupling among most of the neighbor Fe ions is of the superexchange type with an oxygen atom involved in the Fe–O–Fe bond.

The main magnetic properties of SFO, that is an MCA energy of 1.9 meV at 100 K (or 1.4 meV at 300 K) and a Néel temperature of $T_N=780$ K, have been measured decades ago^2^. Still, important properties such as the electronic gap have not yet been determined. In the last 2 decades, significant effort has been devoted to improve its magnetic performance, particularly for permanent magnet applications, by chemical substitution and nanostructuring^3–7^. Nanometric thin SFO platelets have been recently characterized combining fundamental experimental techniques, as X-ray absortion based and Mössbauer spectroscopies, to density functional theory (DFT) calculations intended to rationalize Oxygen *K* absortion edge spectrum measurements^8^. Still, hardly any recent works have revisited the SFO magnetic structure in depth employing state-of-the-art experimental techniques. From the theoretical side, and since the early work of Fang et al.^9^ where Gorter’s prediction^10^ on the ferrimagnetic arrangement of the Fe sublattices was confirmed via ab initio calculations, M-type hexaferrites (M = Ba,Sr) have been extensively studied^11–18^ A number of properties have been derived in these works including, among others, the electronic gap, the saturation magnetization ($M_s$) and the MMs on the Fe ions together with their associated spin-resolved density of states (DOS), the MCA^13,15,16^ as well as exchange couplings (*Js*) subsequently employed in the estimation of $T_N$^12,19^ or the evolution of $M_s$ with temperature^14^. In addition, a large effort has been put in exploring substitutional elements either for the M-ion (La^11,15,19^, Pb^17^, Pr^15^, Nd^15^) or the Fe ions (Al^16^, Zn–Sn^13^) in order to improve the material’s magnetic performance. Most of these studies have relied on the DFT + U framework, paying special attention to the precise value of the Hubbard term U, for which values in the 3–10 eV range have been considered. It is surprising, however, that the simultaneous influence of U on $M_s$, the MCA and $T_N$ has not been addressed systematically in order to derive a unique value that can accurately describe the most relevant SFO’s magnetic properties. In this work we fill this gap performing a comprehensive characterization of the electronic and magnetic properties of SFO at the DFT + U level, providing estimates for the electronic gap, MMs, orbital angular momenta, MCA, *Js* and additionally, within a multi-scale spirit, the $T_N$, here obtained via Monte-Carlo simulations using as input solely the DFT-derived parameters. Moreover, and apart from a systematic study of the Hubbard term U, further attention is paid to physically relevant calculation parameters such as the precise lattice constant, the inclusion of spin–orbit coupling or the ions spin relaxation time in the evaluation of the exchange constants. It turns out that, by far, the most relevant parameter is U, for which a moderate value of 2–3 eV provides excellent agreement with all the available data. Our results are at contrast with similar studies on Ba-hexaferrite^12,19^ where anomalously large U values of 6–10 eV were required to obtain, via the random phase approximation (RPA) or a mean field approach, $T_N$ temperatures close to the experimental ones.

## Results and discussion

### Projected charges and magnetic moments

**Figure 2:**
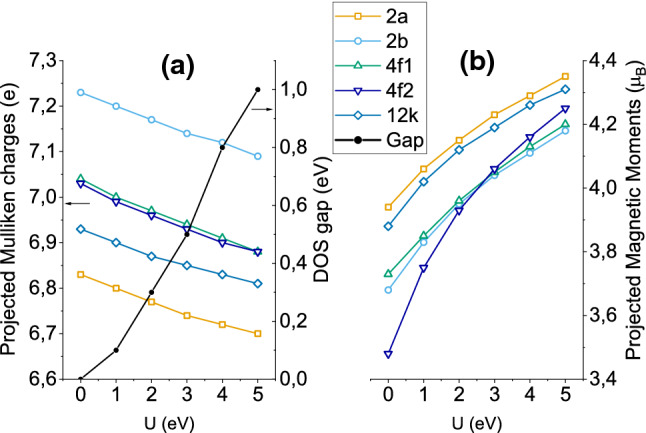
Evolution with the Hubbard term U of: (**a**) the Mulliken charges and, (**b**) the absolute value of the MMs, projected on the Fe sublattices. In (**a**) the dark line shows additionally the calculated SFO band gap (right *y*-axis).

The effect of the Hubbard term on the electronic and magnetic properties projected on each sublattice for the ground state spin configuration is first explored. Figure 2a shows the dependence of the Mulliken charges with U. Although the Mulliken population analysis, being basis dependent, is known to be imprecise when estimating projected charges on ions, it nevertheless provides reliable trends. Here, the non-distorted octahedral Fe-ions (sublattices 2*a* and 12*k*) become more ionized (hold less electronic charge) than the 4*f*1 and 4*f*2 ones which attain almost the same charges despite having different number of oxygen neighbors, while the bipyramidal 2*b* is clearly the less ionized one. All charges diminish with U in the same way (around 0.1*e* loss in the entire U range), implying that the Hubbard term leads to a slightly more ionic character of the Fe–O bonds. In the same figure the evolution of the band gap is shown as well (dark line). As expected, it increases considerably with U, starting from an almost gapless situation (U = 0) and reaching a value of 1.0 eV for ${\hbox {U}}=5$ eV. These values are smaller than the reported SFO gaps in previous DFT + U works which, for ${\hbox {U}} \approx 3$ eV, are in the 0.7–1.0 eV range^11,13,17^ versus the 0.5 eV found here. The discrepancy may be assigned to the tendency of the Siesta formalism to underestimate gaps in semiconductor and insulators probably due to the finite basis set employed. Unfortunately, there is no reported experimental value of the SFO band gap which could be directly compared with the calculated data and, hence, help in the determination of an optimum U.

**Figure 3:**
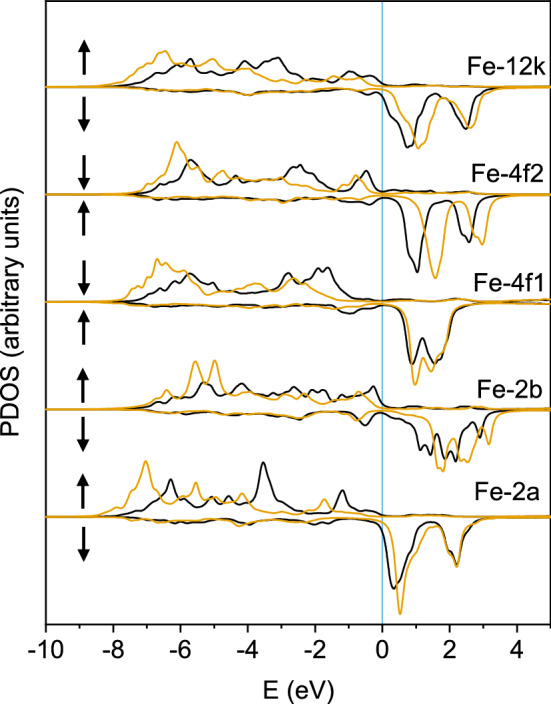
Spin-resolved PDOS for the five Fe sublattices computed at two different U values: 0 eV (black lines) and 3 eV (yellow). For each sublattice the majority spin component along the positve *y*-axis and the minority along the negative *y*-axis are plotted. Notice that since the 4*f*1 and 4*f*2 sublattices are antiferromagnetically coupled to the rest, their up and down spin directions (indicated by arrows at the left of each plot) are interchanged.

Figure 2b displays the MMs per Fe sublattice as a function of U. Roughly, the behaviour of the MMs is anti-correlated with that of the projected charges; the formers increase with U in a non-linear way whith sublattices 2*a* and 12*k* presenting the largest MMs. The increase in the MMs in the ${\hbox {U}} =0$–5 eV interval ($\sim 0.5\,\mu _B$) is similar for all ions except for the anti-ferromagnetically coupled 4*f*2 sublattice, which shows a much larger increase of around $0.8\,\mu _B$. To understand this behaviour, Fig. 3 shows the spin-resolved density of states projected (PDOS) on the iron atoms for ${\hbox {U}}=0$ eV (black lines) and 3 eV (yellow). Apart from the opening of the gap, the main effect of the Hubbard term is a clear shift towards lower energies for the majority components and towards positive energies for the minority electrons, hence yielding a net increase of the MMs of all ions. The largest shift occurs for the minority PDOS of the 4*f*2 ions, which explains their stronger dependence with U. Upon comparison of the calculated MMs with those reported by Liyanage et al.^13^, which may be considered as the most accurate ones as they were obtained under a hybrid exchange-correlation functional free of the Hubbard parameter, an excellent agreement (within $0.06\,\mu _B$) is obtained for all sublattices for the U = 3 eV case (that is, close to the U = 3.7 eV deduced in Ref.^13^).

In summary, increasing U leads to larger magnetic moments, a larger gap and slightly more ionized bonds, which is fully compatible with the fact that the Hubbard term induces a higher level of localization in the Fe-*d* states.

### Exchange couplings

The SFO exchange constants have been obtained from Eq. (2) employing up to 22 different spin configurations and systematically varying the U term in the 2–5 eV interval. In order to check the robustness of our approach, two different unit cell volumes have been considered (the experimental (E) and the theoretically optimized (T) ones) as well as the static (S) and dynamical (D) limits concerning the response time of the ions’ positions to an inversion of their MMs, as explained in section Methods. Throughout this subsection, the calculation conditions used for each set of *J*s will be denoted by two capital letters: ES, ED, TS or TD (for instance, ED refers to *J*s calculated employing the experimental lattice constants under the dynamic limit).

**Figure 4:**
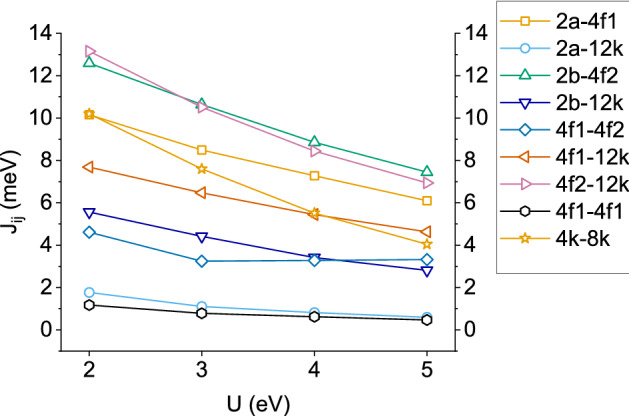
Dependence of the most relevant exchange coupling constants, $J_{ij}$, on the Hubbard parameter U for the ES case.

Figure 4 presents the dependence of the most relevant exchange constants on U for the ES case (the same trend is found for the other three cases). A strong decay in the strength of almost all interactions is apparent as U increases, which is in line with the results of Wu et al.^19^ and Novak et al.^12^ for Ba hexaferrite and derives from the fact that the Hubbard term tends to localize the *d*-states at the Fe sites and hence, their MMs become less influenced by the precise magnetic state of the neighboring ions. Furthermore, all *J*s remain positive indicating a robust anti-ferromagnetic character. The most relevant constants are $J_{4f2-12k}$ and $J_{2b-4f2}$, which attain values over 12 meV at U = 2 eV and decrease to around 8 meV at the largest U. $J_{4f2-12k}$ and $J_{4k-12k}$ are the next in relevance ($J\approx 10$ meV for U = 2 eV), with the latter showing the largest decrease with U as its initial value ends up reduced by $\sim 50\%$. The $J_{2b-12k}$ and $J_{4f1-4f2}$ interactions take intermediate values between 3 and 6 meV, with the latter showing an *anomalous* behaviour for ${\hbox {U}} > 3$ eV as the slope becomes slightly positive. Finally, $J_{2a-12k}$ and $J_{4f1-4f1}$ attain small values between 1–2 eV. The rest of couplings, namely $J_{2a-2b}$, $J_{2a-4f2}$, $J_{2b-4f1}$ and $J_{4f2-4f2}$, have been omitted since they attained almost negligible values below 1 meV in all cases considered.

**Figure 5:**
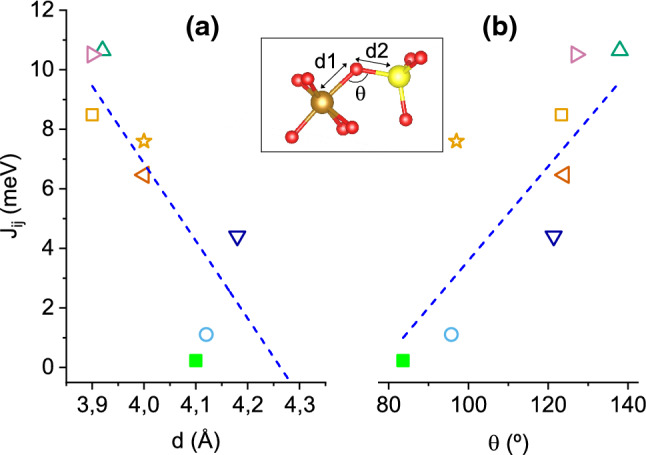
Dependence of the exchange constants strength, $J_{ij}$, on: (**a**) the total Fe–O–Fe bond distance, $d=d_1+d_2$, and, (**b**) the super-exchange Fe–O–Fe angle $\theta$ (see inset). Each exchange constant is represented by the same symbol as that used in Fig. 4, while all values correspond to the ES and U = 3 eV case. In both plots the blue dashed line is a guide to the eye.

In order to rationalize the hierarchy among the calculated *J*s, it should be first noted that their strengths do not correlate with the inverse of the direct Fe–Fe distances (see Table 1). Instead, and as expected for a super-exchange type coupling, it is the angles, $\theta$, and distances, *d*, of the Fe–O–Fe bonds what determine their relative strengths. Figure 5a shows the value of the couplings as a function of the Fe–O–Fe bond distance for the ES case and U = 3 eV—only the eight Fe–Fe couplings mediated by an oxygen atom are included. There is an obvious correlation with the bond distances, as all *J*s with $d<4~\AA$ attain values above 6 meV, while beyond this distance they become clearly smaller. The dependence of the same constants on $\theta$ is displayed in Fig.e 5b. Overall, small *J* values correspond to angles $\theta < 110^\circ$ while almost all of the larger *J*s appear at greater angles. This behaviour corroborates the Goodenough–Kanamori–Anderson (GKA) rules^21,22^, which dictate that the $180^\circ$ super-exchange interaction with partially filled *d* orbitals is strongly antiferromagnetic, while $90^\circ$ bonds tend to be weaker (and sometimes even ferromagnetic). Here, a clear trend towards stronger anti-ferromagnetic coupling is obtained as $\theta$ approaches $180^\circ$ . The rest of interactions which do not involve an oxygen atom attain values below 1 meV with the only exception of $J_{4f1-4f2}$, indicating that this is the only relevant direct exchange term in the SFO.

**Figure 6:**
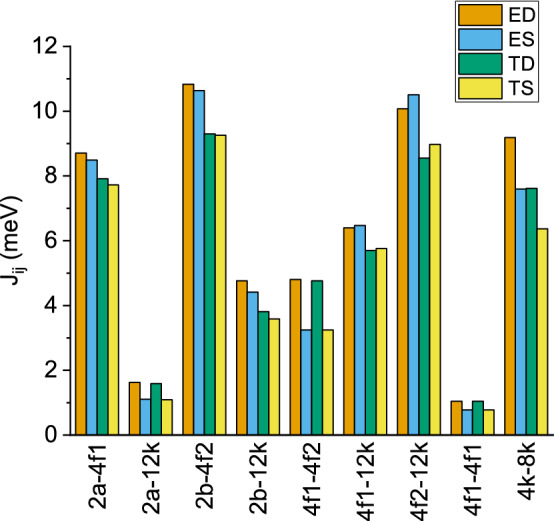
Comparison between the exchange constants obtained under different calculation conditions: experimental lattice under the static (ES) and dynamic (ED) limits, as well as theoretically optimized lattice under the static (TS) and dynamic (TD) limits. All results correspond to the case U = 3 eV.

The effect of varying the lattice constant or relaxing each spin configuration is shown in Fig. 6, where the *J*s corresponding to the ED, ES, TD and TS calculation conditions are displayed side by side, all obtained for a Hubbard term of U = 3 eV (equivalent trends are found for the U = 2 eV and U = 5 eV cases). Two main general conclusions become apparent from this comparison: (1) the expanded theoretical unit cell yields smaller exchange constants by 10% than the experimental one, which is easily understood from the above discussion due to the increase of the Fe–O–Fe bond distances and, (2) the dynamical approach tends to provide slightly larger *J*s for the two lattice constants. The explanation to this latter effect is more subtle and derives from the balance in Eq. (2) between the energy gain per unit cell, $\Delta E$, upon relaxation of spin configurations *i* plus *j* versus that of the combined spin configuration *ij*. The results indicate that $\Delta E_i+\Delta E_j$ tends to be larger than $\Delta E_{ij}$, although this is not a general rule and some exceptions can be seen in the figure (for instance, the $4f1-12k$ and $4f2-12k$ interactions).

Finally, the influence of the inclusion of the spin-orbit interactions on the exchange couplings has been examined for some selected cases. However, for the largest *J*s the difference was only $\sim \, 0.2\%$, indicating that SOC may be safely ignored when computing the exchange constants. This is not surprising since energy differences between different spin configurations are typically of the order of eVs, while SOC contributions are at least one order of magnitude smaller.

### Néel temperature, $T_N$

Once the magnetic moments and exchange constants under different calculation conditions have been obtained, the SFO’s Néel temperature, $T_N$, may be estimated following two different approaches: (1) analytically using the Generalized Molecular Field Theory (GMFT) and, (2) numerically via Monte Carlo simulations (see section “Methods”).

**Figure 7:**
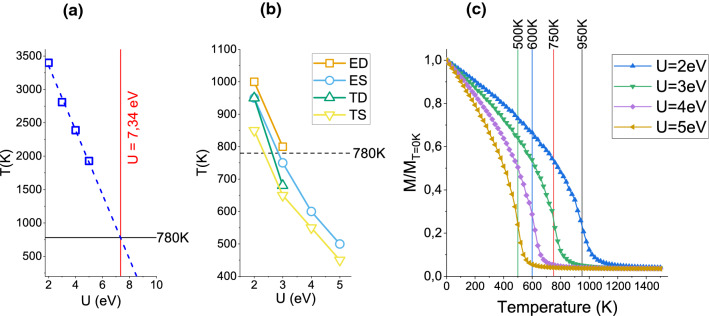
Néel temperature as a function of the U parameter obtained from: (**a**) the GMFT approximation (blue squares) and, (**b**) Monte Carlo simulations for the ES, TS, ED and TD calculation conditions. The horizontal dashed line indicates the experimental $T_N^{exp}$ value in both panels. In (**a**) the blue dashed line is a linear fit to the data points. (**c**) Total magnetization of the unit cell normalized to that at $T=0$ as a function of the system temperature in the Monte Carlo simulations. Data obtained from the ES exchange constants and for different U values. The Néel temperature, $T_N$, in each case is indicated by the vertical lines.

Figure 7a shows, as a function of U, the $T_N$s derived from the GMFT formalism after employing the DFT derived $J_{ij}$ constants (ES case) in expression (6). The graph shows a clear linear dependence with a strong decrease of the temperatures as U increases. However, even at the largest U value considered, $T_N$ is close to 2000 K, that is, more than twice the experimental value, $T_N^{exp}=780$ K. In fact, by linear extrapolation $T_N^{exp}$ would be recovered at a U value as large as 7.3 eV. It should be recalled that a mean field approach was also employed in Refs.^12,19^ and, similar to our case, a best fit to the experimental Néel temperature could only be obtained for a large Hubbard term ${\hbox {U}} \ge 7$ eV—inclusion of spin fluctuations at the RPA level reduced the $T_N$ overestimation but, still, yielded optimized U values of around 7 eV.

Panel (b) in Fig. 7 displays the Néel temperatures obtained from the Monte Carlo simulations as a function of U and for the four calculation conditions described above. Plots of the evolution of the cell magnetization with the system temperature, from which the $T_N$ values are deduced, are presented in panel (c). Although $T_N$ also decreases as U is increased, this time all curves reach values close to $T_N^{exp}$ in a narrow ${\hbox {U}}= 2.5$–3.2 eV range, with the ED and ES cases yielding slightly larger critical temperatures (for fixed U) than the TD and TS ones, as expected from the similar behaviour followed by the exchange constants (Fig. 6). The main conclusion, therefore, is that the Monte Carlo approach results more reliable than the GMFT, as it leads to an *optimized* Hubbard term clearly smaller than that derived from the GMFT, $\sim \, 3$ eV versus $\sim \, 7$ eV, in better agreement with the 3.7 eV deduced in Ref.^13^ as well as with a number of DFT + U calculations involving Fe ions under different chemical environments^23–26^.

### Magnetic anisotropy and orbital angular momenta

**Figure 8:**
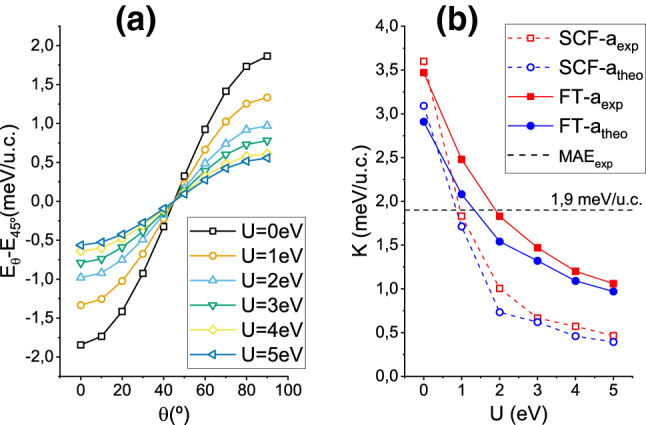
(**a**) Total energies per unit cell (u.c.), $E_\theta$, obtained under the FT approach for different polar angles of the spin quantization axis ($\theta =0$ corresponds to out-of-plane magnetization and $\theta =90^\circ$ to in-plane). All curves have been substracted by their value at $\theta =45^\circ$ for visual purposes. The MCA in each case is given by the difference $K=E_{0^\circ } - E_{90^\circ }$. (**b**) Evolution of the MCAs with the Hubbard term U calculated for both the experimental and theoretically optimized lattice constants ${\hbox {a}}_{\mathrm {exp}}$ and ${\hbox {a}}_{\mathrm {theo}}$, respectively. SOC has been included either at the FT level (solid lines) or self-consistently (dashed lines). The dashed horizontal line corresponds to the experimental MCA energy per unit cell.

In this section magnetic properties which require the inclusion of SOC in the DFT+U calculations are addressed (see section “Methods”). Focusing first on the magnetic anisotropies, Fig. 8a shows the evolution of the SFO’s total energy per unit cell, $E_\theta$, as the spin quantization axis is rotated from the out-of-plane (0001) direction ($\theta =0$) to the in-plane (1000) direction ($\theta =90^\circ$) and for the different U values considered. In these calculations, evaluated at the experimental lattice constant, the SOC has been included at the level of the force theorem (FT) approximation. All curves show a nice $K \sin ^2\theta$ behavior, with a marked perpendicular magnetic anisotropy (PMA), although it is steadily reduced as U is increased. The associated MCAs, defined as the difference $K=E_{90^\circ } - E_{0^\circ }$, are displayed in Fig. 8b, where the evolution of the PMA is plotted as a function of U and for both the experimental and theoretical lattices (solid red and blue lines, respectively). For a fixed U value, the PMAs calculated at the experimental lattice constant are always larger than those obtained using the theoretical lattice. Naively, the decrease of the PMAs with increasing unit cell volume or increasing U may be understood from the reduction of the effective crystal field due to either smaller Fe–O overlaps or an increased electron localization. Nevertheless, the most relevant conclusion in Fig. 8b is that, for the experimental lattice constant, the low temperature experimental PMA of 1.9 meV^2^ is retrieved for ${\hbox {U}} \approx 2$ eV, which is close to the optimum 3 eV value deduced in the previous subsection. Notice that the fitted U in the figure approaches 3 eV if the 300 K experimental PMA value of 1.4 meV is considered instead.

In the same figure the MCAs calculated self-consistently, that is, beyond the FT approximation, are also shown by dashed lines. They present the same trend with U as their FT counterparts, but attain smaller values (by more than 0.5 meV) which are in closer agreement with previous MCA calculations on M-hexaferrites where PMAs of around 0.8 eV were reported for U in the 3.7–4.5 eV range^13,15,16^. As a consequence, the experimental MCA energy is retrieved at unsually small values of ${\hbox {U}} \approx 1.0$–1.5 eV. Nevertheless, we consider more reliable the MCAs derived from the FT given their nice $\sin ^2\theta$ behavior for all U values [panel (a)] while the self-consistent approach failed to yield such smooth MCA($\theta$) curves for finite U—mainly due to numerical instabilities in the precise occupation number of the Fe-3*d* states. Furthermore, and as expected from the relatively small SOC of the Fe ions, the FT reproduces fairly well the self-consistent values in the absence of the Hubbard term (U = 0).

**Figure 9:**
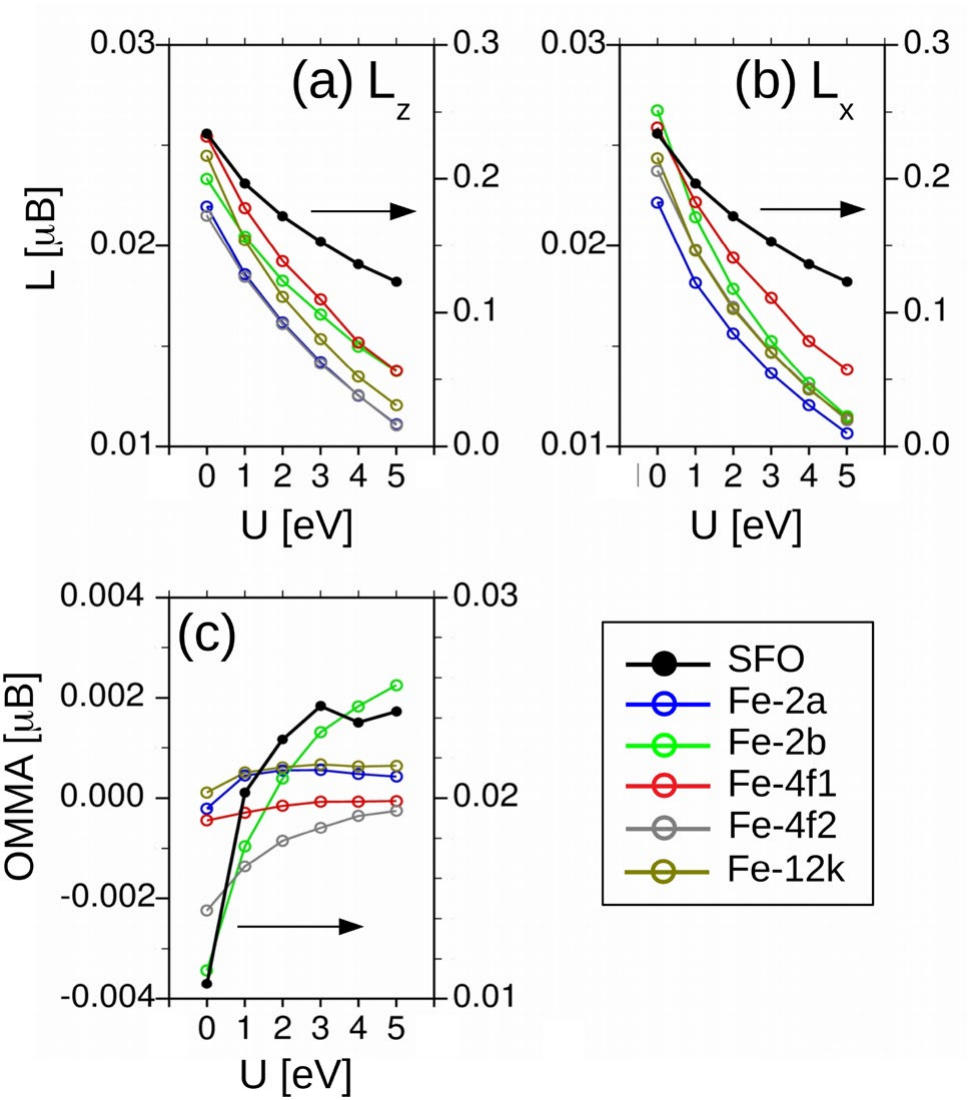
OMMs for the SFO unit cell (black line and right axis) and for each Fe ion (colored lines and left axis) for: (**a**) out-of-plane and, (**b**) in-plane spin quantization axis. In both plots the sign of the antiferromagnetically coupled 4*f*1 and 4*f*2 sublattices has been inverted for visualization purposes. (**c**) Orbital magnetic moment anisotropy, ${\hbox {OMMA}}=L_z-L_x$, for the SFO and each of the Fe sublattices. Here the signs of the 4*f*1 and 4*f*2 ions have not been inverted, so that their negative values correspond to an out-of-plane OMMA.

Last, Fig. 9 shows the orbital magnetic momenta (OMM) in absolute value calculated for the experimental lattice and including SOC self-consistently for the spin quantization axis aligned out-of-plane, panel (a), and in-plane, panel (b). The net OMM of the SFO unit cell (black lines and right axis) attains a considerable value of $0.25\,\mu _B$ at U = 0, but decreases non-linearly as U increases down to $\sim 0.13\mu _B$ at U = 5 eV. The OMMs of the individual Fe-ions (colored lines and left axis) are an order of magnitude smaller; still, they also follow the same trend with U (recall that the sign of the OMMs for the 4*f*1 and 4*f*2 ions appears inverted in the graph). Panel (c) displays the orbital magnetic momenta anisotropy (OMMA) defined as $L_z - L_x$ (here the sign of the 4*f*1 and 4*f*2 ions has not been inverted). The OMMA of the SFO remains out-of-plane but, unexpectedly, it increases with the Hubbard strength—we associate the small kink at U = 4 eV to numerical inaccuracies—as a result of a stronger attenuation with U of the out-of-plane OMM. A similar behaviour is found for all individual ions, with the peculiarity that the 2*a* and specially the 2*b* sublattices show an in-plane OMMA at small U values. It is timely to note that the opposite dependence on U between the OMMA and the MCA points to the fact that the correlation between the two quantities is far from trivial, as it is often assumed in perturbative theoretical approaches^27,28^.

## Conclusions

A comprehensive study at the DFT + U level of the magnetic properties of the strontium hexaferrite (SFO) has been performed, providing estimates of the Fe-resolved spin and orbital magnetic moments, the MCAs and the associated exchange constants. Furthermore, the influence of different calculation parameters on these properties, such as the Hubbard strength, the precise lattice parameters or the relaxation time of the ions versus spin relaxations (static or dynamic limits) has been explored in detail.

It has been found that, by far, the most relevant parameter is U, as it progressively induces a larger electron localization around the ions which translates into larger gaps and MMs, smaller *J*s and smaller MCAs and OMMs (but, surprisingly, larger OMM anisotropies). Rather than a drawback, such a strong dependence may be considered as an excellent opportunity for deriving an optimium U value for 3*d* electrons in different magnetic systems. Here, a value of ${\hbox {U}} \approx 2$–3 eV nicely reproduces the experimental MCA and yields exchange constants which, when employed in micro-magnetic Monte Carlo simulations, accurately reproduce the SFO’s Néel temperature as well.

We note that it is common practice within DFT + U studies to treat the Hubbard strength as an adjustable parameter; its value is tpycally tuned to obtain specific electronic (mainly the gap) or thermodynamic properties^24,26,29–31^. However, and apart from atomic MMs, magnetic properties such as the MCA or the critical temperature are rarely employed as targets^19,32^ in the fitting process. In this work it has been shown that from the simultaneous fit to both quantities, meaningful U strengths are obtained which can ultimately deliver reliable ab initio-derived parameters for use in micromagnetic simulations.

## Methods

All ab initio calculations have been performed with the Green code^33,34^ and its interface to the DFT-pseudopotential Siesta package^35^. In a first stage both the Local Density (LDA) and the Generalized Gradient^36^ (GGA) approximations were considered for the exchange-correlation functional, although results will only be presented for the latter since LDA consistently provided larger structural deviations from the experimental SFO lattice parameters, as well as it often led to spurious low-spin states for the Fe ions so that, overall, it may be considered less reliable. Hubbard type corrections were included within the DFT+U formalism following the Dudarev approach^29,37,38^. For the single parameter U different values were considered while its effect on the computed SFO properties is extensively discussed in the Results section.

A double-zeta polarized (DZP) atomic orbital (AO) basis set was defined for all elements. The AOs are strictly localized as determined from a confinement energy of 100 meV. Pseudo-core corrections were included for both the Fe and Sr ions. The resolution of the real space grid was set to an ultra-fine value below $0.04~{\AA }^3$ (equivalent to a Mesh Cutoff of 2000 Ryd) to ensure a good convergence around the Fe cores, while the reciprocal space was sampled with a $(9\times 9\times 3)$ supercell. A value of 100 meV was used in the Fermi–Dirac distribution function (electronic temperature).

In the DFT models both the experimental SFO lattice constants ($a=b=5.884~\AA$ and $c=23.05~\AA$) as well as the theoretically GGA optimized values ($a=b=5.955~\AA$ and $c=23.33~\AA$) were considered. For both cases, in the determination of the SFO ground state all ions were allowed to relax until forces on atoms were below 0.02 eV/Å.

For those calculations including spin–orbit coupling (SOC) the fully relativistic pseudopotential (FR-PP) approach^38,39^ was used to construct the SOC Hamiltonian. MCAs and orbital magnetic moments on the Fe-ions were calculated self-consistently varying the spin quantizaton axis from the out-of-plane to in-plane direction, $\theta =0^\circ$ and $90^\circ$, respectively. Additionally, an alternative estimation of the MCAs was obtained via the force theorem (FT) based on the difference in the non-self-consistent band energies between different spin quantization axis^40,41^. Comparison between both approaches yielded a good agreement in the absence of the Hubbard term, in accordance with previous MCA studies under the same formalism^41^. However, deviations of 0.6–0.8 meV were found for finite U values, as discussed in the main text and shown in Fig. 8.

Exchange constants, $J_{ij}$, between sublattices *i* and *j*, have been calculated from the energy differences between different spin-collinear configurations. To this end, and starting from the usual Heisenberg Hamiltonian, the exchange energy for a given spin configuration $\alpha$, is expressed as^19,42^:

1 $$\begin{aligned} E_\alpha = -\frac{1}{2} \sum _{i,j}^{N} n_i z_{ij} J_{ij} \; \mathbf {S}^\alpha _i \cdot \mathbf {S}^\alpha _j \end{aligned}$$

where $n_i$ is the number of atoms in the *i*-th sublattice, $z_{ij}$ the number of nearest neighbors to an *i*-th ion that belong to sublattice *j*, and $\mathbf {S}^\alpha _{i/j}$ are the spin vectors of the ions in the *i*/*j* sublattice.

Based on Eq. (1), and assuming that the modulus $S_i$ of the spin vector $\mathbf {S}^\alpha _i$ of the *i*-th ions does not change between different spin configurations (that is, independent of $\alpha$), it is straigthforward to show that the exchange constants $J_{ij}$ between sublattices *i* and *j* can be directly determined from the expression^19^:

2 $$\begin{aligned} J_{ij}= \frac{ E_{ij} + E_0 - E_i - E_j }{4n_iz_{ij}\; \mathbf {S}_i^0 \cdot \mathbf {S}_j^0 } \end{aligned}$$

where $E_0$ corresponds to the DFT total energy of the ground state spin configuration $\alpha =0$, $E_{i/j}$ to that of the spin configuration where the spin of sublattice *i*/*j* has been inverted with respect to 0, and $E_{ij}$ to that where the spins of both lattices *i* and *j* have been simultaneously flipped.

The number of neighbours and their distances between all SFO sublattices are provided in Table 1. As the exchange interaction decreases with the distance, only first nearest neighbours need to be considered^19^. In order to determine the intra-sublattice exhange couplings, the *4f1*, *4f2* and *12k* groups have been splitted into sub-sublattices *4f1a-4f1b*, *4f2a-4f2b* and *4k-8k*, respectively. The other two intra-sublattice couplings, *2a-2a* and *2b-2b*, may be safely ignored due to their large nearest neighbor distances (see Table 1). This leads to 13 exchange constants for this material: $J_{2a-2b}$, $J_{2a-4f1}$, $J_{2a-4f2}$, $J_{2a-12k}$, $J_{2b-4f1}$, $J_{2b-4f2}$, $J_{2b-12k}$, $J_{4f1-4f2}$, $J_{4f1-12k}$, $J_{4f2-12k}$, $J_{4f1-4f1}$, $J_{4f2-4f2}$, $J_{4k-8k}$ which, according to Eq. (2), require up to 21 different spin configurations apart from the ground state.

**Table 1:** Nearest neighbours distances in Å and number of nearest neighbor ions, $z_{ij}$, in parenthesis, for the five Fe-sublattices, obtained for the theoretically optimized SFO lattice constants.

	2*a*	2*b*	4*f*1	4*f*2	12*k*
2*a*	5.95 (6)	5.84 (2)	3.50 (6)	5.62 (6)	3.09 (6)
2*b*		5.95 (6)	6.23 (6)	3.71 (6)	3.72 (6)
4*f*1			3.66 (3)	3.82 (1)	$\sim$3.56 (9)
4*f*2				2.77 (1)	3.53 (6)
12*k*					$\sim$2.98 (4)

A systematic study of the influence of U on the $J_{ij}$ constants has been performed via DFT calculations for the 22 spin configurations varying U between 0 and 5 eV in 1 eV steps. These sets of calculations have been done for both the experimental and the theoretically optimized lattice constants. Furthermore, the exchange constants have been computed under two different limits. In a first stage the optimized ground state geometry (calculated independently for each U value) was used for all spin configurations $\alpha$. Such standarized approach assumes that the spin orientation on the atoms evolves in time much faster than the ions themselves move as a consequence of the *exchange* forces. Thus, it is denoted as the *static* limit. In a second stage the SFO geometry was optimized for each magnetic configuration independently, thus simulating a *dynamic* limit where the spin relaxation time would be much longer than the atoms response to the exchange forces.

**Figure 10:**
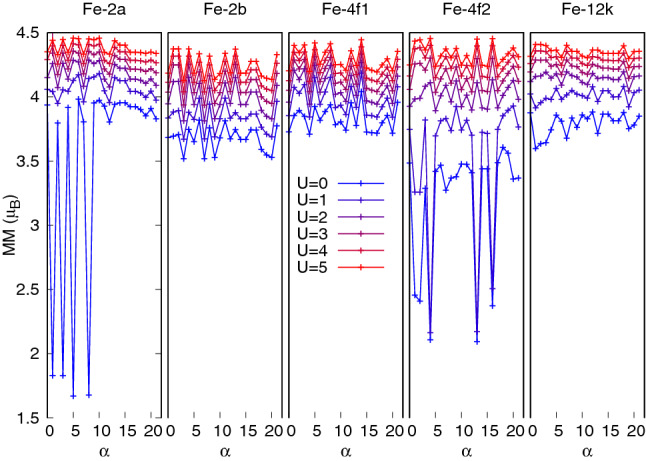
MMs for each Fe sublattice as a function of the spin configuration index $\alpha$ (the case $\alpha =0$ corresponds to the ground state). Each color corresponds to a different value of U, as indicated in the legend in eV. The MMs in sublattices 2*a* and 4*f*2 attain anomalously small values (low spin states) when ${\hbox {U}} \le 1$ eV at some specific spin configurations.

It should be noted, however, that the assumption in Eq. (2) that the modulus of $\mathbf {S}^\alpha _i$ does not change among spin configurations was not fullfilled in certain cases for ${\hbox {U}} \le 1$ eV. Figure 10 provides a summary of the MMs per Fe sublattice for the 22 spin configurations and all U values considered. Although most of the MMs remain fairly constant (within less than $0.5~\mu _B$) for all $\alpha$, at small U values sublattices 2*a* and 4*f*2 suffer a drop of their MMs to low spin states in a few spin configurations, thus invalidating the estimation of the *J*s via Eq. (2). Therefore, results of the exchange constants are presented for values of ${\hbox {U}} >1$ eV only. Recall, however, that no low spin-states appear for the ground state (case $\alpha =0$) in any of the sublattices throughout the entire U range.

Micro-magnetic Monte Carlo simulations have been performed within the software Vampire^43^ using our DFT + U results (exchange constants and MMs) as inputs to estimate the Néel temperature, ${\hbox {T}}_N$, of the SFO. A $(5\times 5\times 5)$ supercell comprising only Fe sub-lattices was employed in these calculations (leading to a total of 3000 atoms). The temperature of the system was slowly increased from 0 K up to 1500 K in 20 K steps. Each temperature step included 50,000 equilibration cycles followed by another 50,000 time steps.

Alternative, less accurate, estimations of $T_N$ have been performed under the Generalized Molecular Field Theory (GMFT), which represents a generalization of Weiss MFT to high order and anisotropic interactions^44,45^. Within this theory, the so called Weiss molecular field acting on the atoms of the *i*-th sublattice, $H_i$, is given by:

3 $$\begin{aligned} H_i = H_0 + \sum _{j=1}^{n}\gamma _{ij}M_j \end{aligned}$$

where $H_0$ is the external magnetic field, $M_j$ the thermal averaged MM of the *j*th sublattice, defined as $M_i=g\mu _B\langle S_i\rangle$, where $S_i$ is the spin of the atoms of sublattice *i* and *g* is the Landé factor. $\gamma _{ij}$ are the molecular field coefficients for a field exerted on an *i*-th sublattice atom by a neighbor atom of the *j*-th sublattice: $\gamma _{ij} = 2z_{ij}J_{ij}/(N_jg^2\mu _B^2)$, with $z_{ij}$ the number of *j* neighbors of an *i* atom, $J_{ij}$ the exchange constant between *i* and *j* sublattices (here derived from the DFT calculations) and $N_i$ the number of atoms at the $i-$th sublattice. Note that the $\gamma _{ij}$ coefficients are symmetric and it is typical practice to set the diagonal terms $\gamma _{ii}=0$. For our SFO system, this requires to remove any intra-sublattice interactions by splitting sublattice 12*k* into 3 different sub-sublattices each with four atoms.

The main idea behind the GMFT is to assume that the material behaves like a paramagnet at high enough temperatures well above the magnetic ordering temperature ($T_N$ for ferrimagnets), in which case the magnetization may be expressed as:

4 $$\begin{aligned} M_i = N_ig\mu _B S_i B_S \left( \frac{g\mu _B H_i}{k_BT}S_i\right) \end{aligned}$$

where $B_S$ are the Brillouin functions. For temperatures sufficiently high, these functions can be approximated by $B_S(x) = \frac{S+1}{3S}x$, so one ends up with a system of linear equations:

5 $$\begin{aligned} M_i = N_ig\mu _B S_i\frac{S_i+1}{3S_i}\frac{g\mu _B H_i}{k_BT}S_i = \frac{C_i}{T}H_i = \frac{C_i}{T}(H_0+\sum _{j=1}^{n}\gamma _{ij}M_j) \end{aligned}$$

In order to calculate the critical temperature for the onset of paramagnetism, the external field is removed ($H_0=0$), and the non-trivial solutions to the above system of equations are obtained by setting the associated determinant to zero:

6 $$\begin{aligned} \begin{gathered} \begin{vmatrix} \frac{T}{C_1}&-\gamma _{12}&\ldots&-\gamma _{1n} \\ -\gamma _{12}&\frac{T}{C_2}&\ldots&-\gamma _{2n} \\&\ddots \\ -\gamma _{n1}&-\gamma _{n2}&\ldots&\frac{T}{C_n} \end{vmatrix} =0 \end{gathered} \end{aligned}$$

In the SFO case this leads to a $7\times 7$ determinant that leads to 7 different critical temperatures, each of them associated with a transition to a specific spin arrangement. The one with the highest value corresponds to the Néel temperature, $T_N$.
